# The co‐adaptation of a lifestyle program to improve sleep in persons living with dementia

**DOI:** 10.1002/alz70858_105549

**Published:** 2025-12-26

**Authors:** Tara Kuhn, Carrie A McAiney, Andrew Lim, Laura E. Middleton

**Affiliations:** ^1^ University of Waterloo, Waterloo, ON, Canada; ^2^ University of Toronto, Toronto, ON, Canada

## Abstract

**Background:**

Sleep disturbance is a common experience in persons with Alzheimer's disease (PWAD) and can be stressful for care partners to manage. However, few programs have been designed to support sleep among PWAD. The few existing programs may not be appropriate for all contexts (e.g., geography, weather). Using co‐design to develop or adapt interventions can help create programs that identify and overcome local barriers to programming.

**Method:**

The *Nighttime Insomnia and Treatment Education for Alzheimer's Disease* (NITE‐AD) by McCurry et al. (2005) is a program for care partners, designed to improve sleep of PWAD. Previous offerings of the NITE‐AD program have effectively reduced total awake time at night among PWAD. However, adaptation of program materials and physical activity recommendations was needed for winter use in the Canadian context. The co‐adaptation of the NITE‐AD program (to the *Nighttime Insomnia and Treatment Education for Canadians* *Alzheimer's Disease*, NITE‐CAD program) took place in 5 stages: 1) understanding the barriers, facilitators, and supports needed for winter physical activity among PWAD, 2) engaging a co‐advisory team, 3) co‐adapting the program; and 4) assessing the feasibility of NITE‐CAD.

**Result:**

Winter conditions were a barrier to physical activity, with driving in snow or ice and the extra time needed to prepare for outdoor activities as notable challenges. Some overcame these challenges through specialized equipment and/or accessible facilities. Three persons with lived experience (a care partner, a dementia exercise provider, an Alzheimer Society staff) were recruited to the co‐adaptation team. Program adaptations included: having multiple physical activity options, increasing accessibility of the manual and using Canadian specific resources, and encouraging PWAD to participate in the program. Unfortunately, we were unable to assess the feasibility of NITE‐CAD due to recruitment challenges. Among those who expressed interest, people were not eligible due to high risk of sleep apnea or having dementias other than Alzheimer's disease.

**Conclusion:**

The co‐adaptation of NITE‐CAD identified and implemented changes important to the Canadian, and especially winter, context. However, more work is needed to co‐design recruitment processes and eligibility criteria to ensure that the program is relevant and accessible.

